# Link N Directly Targets IL-1β to Suppress Inflammation and Regulate Sensory Pain in Intervertebral Disc Degeneration

**DOI:** 10.3390/biom15040603

**Published:** 2025-04-19

**Authors:** Michael P. Grant, Muskan Alad, Fajer Yousef, Laura M. Epure, John Antoniou, Fackson Mwale

**Affiliations:** 1 Department of Surgical and Interventional Sciences, McGill University, Montreal, QC H3T 1E2, Canadalaura.epure@mcgill.ca (L.M.E.);; 2 Orthopaedic Research Laboratory, Lady Davis Institute for Medical Research, Montreal, QC H3T 1E2, Canada; 3 SMBD-Jewish General Hospital, McGill University, Montreal, QC H3T 1E2, Canada

**Keywords:** intervertebral disc, degeneration, back pain, link N, interleukin-1β, inflammation

## Abstract

Intervertebral disc (IVD) disease is typically characterized by the degradation of IVD tissue, secretion of inflammatory and painful factors, and hyperinnervation of the disc. The pro-inflammatory cytokine interleukin-1β (IL-1β) has been regarded as a principal factor in orchestrating disc degeneration. Link N (LN) is a peptide derived from the link protein that has been shown to promote extracellular disc regeneration even in an inflammatory milieu; however, no mechanism(s) has been described for their behaviour to date. Building on prior studies on LN, we hypothesize that LN directly inhibits IL-1β. IVD degeneration was experimentally induced in New Zealand white rabbits, followed by the injection of either sLN or saline as the vehicle control. To determine the expression of markers of pain, histology was performed. Cultured human Nucleus Pulposus disc cells (hNP) were used to determine the effects of LN on IL-1β-induced changes in gene expression, including the effects on IL-1β, TNFα, and IL6 signalling. Isolated murine dorsal root ganglia (DRG) neurons were used to assess the effect of LN on IL-1β-induced neuronal hyperactivity. LN significantly reduced IL-1β-induced NF-κB activation in a dose-dependent manner in disc cells and was further able to modulate IL-1β-induced gene expression, inflammatory mediators, and neurotrophic factors. Peptide docking simulations revealed that LN could interact with IL-1β. A direct interaction of LN and IL-1β was revealed through co-immunoprecipitation experiments. Although IL-1β was able to hypersensitize DRG neurons following a seven-day exposure, as demonstrated by Ca^2+^ imaging, this effect was significantly blunted when co-treated with LN. LN demonstrates a novel mechanism of action by directly inhibiting IL-1β, in addition to mitigating IL-1β-induced hypersensitivity in DRG neurons. These data suggest a potential role for LN in reducing discogenic pain.

## 1. Introduction

Chronic low back pain is a leading cause of disability worldwide, with significant socioeconomic and healthcare burdens [1,2,3,4,5]. A major contributor is intervertebral disc (IVD) degeneration, a progressive condition associated with a loss of extracellular matrix (ECM) components, increased cellular apoptosis and senescence, inflammation, and the innervation of nociceptive sensory fibres, ultimately leading to chronic pain and functional impairment [3,4,5]. Despite its high prevalence, no effective medical treatments currently exist to halt or reverse disc degeneration, highlighting the urgent need for novel therapeutics targeting both degeneration and inflammation in the IVD.

The degenerative process in IVD degeneration is a complex, multifactorial cascade involving biochemical, structural, and cellular changes that compromise disc integrity and function [6,7,8]. At the molecular level, the inflammatory cytokine interleukin-1β (IL-1β) plays a central role in mediating disc degeneration [2]. IL-1β promotes the expression of matrix-degrading enzymes such as matrix metalloproteinases (MMPs) and aggrecanases, creating a pro-inflammatory microenvironment that exacerbates tissue breakdown while suppressing the production of ECM components, including proteoglycans and type II collagen [9]. Additionally, IL-1β drives the innervation and vascularization of degenerative discs through the upregulation of neurotrophic factors like nerve growth factor (NGF) and brain-derived neurotrophic factor (BDNF) [2,6,10], which bind their cognate receptors, TrkA and TrkB [2,9,10,11], to stimulate sensory nerve growth. This neurogenic response leads to increased nociceptive signalling and peripheral sensitization, exacerbating pain in disc degeneration [2,6]. The dorsal root ganglia (DRG), which house the cell bodies of sensory neurons, play a critical role in transmitting pain signals from degenerative discs to the central nervous system [12]. DRG neurons not only respond to IL-1β but also express both IL-1β and its receptor IL-1R1, suggesting an autocrine–paracrine mode of IL-1β action [13]. The peripheral administration of IL-1β has been shown to activate DRG sensory fibres, leading to hyperalgesia and increased neuronal excitability [14]. Together, these findings highlight IL-1β as a major driver of both tissue degeneration and pain, making it an attractive therapeutic target for IVD degeneration [11,14].

Currently, no therapies exist to simultaneously address the dual pathological processes of ECM degeneration and inflammation-induced pain in IVD [15]. However, recent evidence suggests that Link N (LN), a naturally occurring 16-amino acid peptide derived from the N-terminal region of the link protein, may offer a unique therapeutic approach. The link protein is an essential component of the ECM that stabilizes the interaction between hyaluronic acid and aggrecan, which are both critical for disc hydration and mechanical function. LN is generated through the proteolytic activity of MMPs during normal tissue turnover and has been shown to exert anabolic effects in the IVD by stimulating the synthesis of proteoglycans and type II collagen [16]. Our group previously demonstrated that LN can partially activate Smad1/5 signalling through the bone morphogenetic protein (BMP) receptor pathway, promoting ECM regeneration even in an inflammatory milieu. In a rabbit annular puncture model of IVDD, LN treatment restored disc height and matrix content, underscoring its regenerative potential [17].

Beyond its anabolic properties, emerging evidence suggests that LN may also exhibit anti-inflammatory and anti-nociceptive effects. In a murine model of osteoarthritis, the intra-articular administration of LN significantly reduced pain behaviour within six hours of treatment, independent of structural regeneration. This rapid response indicates that LN may act directly on inflammatory and pain-signalling pathways [18]. In our previous report, we show that LN suppresses the IL-1β-induced upregulation of NGF, BDNF, and their receptors in annulus fibrosus (AF) [19].

This study aims to investigate the ability of LN to modulate IL-1β signalling in NP cells and its impact on IL-1β-induced neuronal hyperactivity in DRG neurons. These findings will provide novel insights into the therapeutic potential of LN as a treatment for disc degeneration and associated chronic back pain, offering a dual-targeted approach, regenerating disc tissue and alleviating pain.

## 2. Materials and Methods

### 2.1. Peptide Synthesis

Link N (LN) (DHLSDNYTLDHDRAIH), Link N-Biotin (LN-B) (DHLSDNYTLDHDRAIH-K-Biotin), scrambled Link N (SC) (DLNRAHLHIDYHTDSD), and scrambled Link N-Biotin (SC-B) (DLNRAHLHIDYHTDSD-K-Biotin) were synthesized with a purity >98% via CanPeptide (Montreal, QC, Canada).

### 2.2. Antibodies

Anti-interleukin-1β antibody was purchased from Thermo Fisher Scientific (Waltham, MA, USA, Cat# MM425B) and the anti-phospho-NF-κB p65 antibody was purchased from Cell Signaling Technology (Danvers, MA, USA). Anti-IL1 Receptor I (anti-IL1R1) was purchased from Abcam (Cambridge, MA, USA, cat# ab106278); monoclonal anti-polyHistidine antibody was purchased from Millipore Sigma (Burlington, MA, USA, cat# H1029). Anti-LN antibody was custom-prepared by Thermo Fisher Scientific in rabbits, using LN as antigen. Anti-Nerve Growth Factor (NGF) antibody was purchased from Abcam (cat# ab52918), including anti-Protein Gene Product 9.5 (PGP9.5) (cat# ab8189), anti-CGRP (cat# ab81887), and anti-aggrecan (cat# ab36861) antibodies.

### 2.3. Human Nucleus Pulposus Cells

Human NP cells (hNP) were purchased from ScienCell Research Laboratories (Carlsbad, CA, USA, cat. #4800). Cells were cultured in PrimeGrowth^®^ Culture Medium (Wisent Bioproducts, Montreal, QC, Canada, cat# 319-510). Different lots were purchased, representing different donors. Medium was replaced every three days. Cells were not used beyond passage four.

### 2.4. RNA Extraction and Quantitative Real-Time PCR

Human NP cell micropellets were cultured for 6 days in 0.5 mL medium supplemented with either IL-1β [5 ng/mL] 3131, LN [1 µg/mL] + IL-1β, LN [10 µg/mL] + IL-1β, or with the vehicle (PBS) alone as a control. The Total RNA minikit (Geneaid Biotech Ltd., New Taipei City, Taiwan) was used to extract RNA following the manufacturer’s instructions. Complementary DNA was synthesized using a SuperScript Vilo cDNA synthesis kit (Thermo Fisher Scientific, Waltham, MA, USA). The quantitative real-time PCR of hNP cells was quantified using an ABI 7500 fast light cycler using CYBR green master mix (Thermo Fisher Scientific, Waltham, MA, USA) and specific primers (Table 1).

Relative mRNA expression levels were normalized against GAPDH [20].

Relative expression = 2^−ΔΔCt^.

### 2.5. Effect of LN on Cytokine Response

Human NP cells were serum-deprived overnight and incubated in culture medium containing IL-1β [5 ng/mL], TNF-α [10 ng/mL], or IL-6 [5 ng/mL], with or without increasing concentrations of LN [0.01, 0.1, 1 or 10 μg/mL], for 5–60 min at 37 °C in serum-free medium, where indicated. Control cells were incubated with serum-free medium for 10 min. Cells were lysed in RIPA (radio immuno-precipitation assay) buffer (Millipore Sigma, cat# R0278) containing protease cocktail II (Sigma-Aldrich, St. Louis, MO, USA) and phosphatase (Thermo Fisher Scientific, Waltham, MA, USA) inhibitors. Western blotting was performed on cell lysates via electrophoresis on 4–20% gradient gels (Bio-Rad, Hercules, CA, USA) under reducing conditions. Lysates were then transferred to 0.2 µm PVDF membranes (BioRad, Hercules, CA, USA, cat# 1620177XTU). Blots were then probed with anti-phospho-NF-κB p65 antibody (Cell Signaling Technology, Danvers, MA, USA) and GAPDH (Sigma-Aldrich, St. Louis, MO, USA) for the normalization of IL-1b and TNF-a, and with anti-phospho-STAT3 antibody (Cell Signaling Technology, Danvers, MA, USA) and GAPDH for the normalization of IL-6. Blots were washed and incubated with Amersham ECl Select Western blotting detection reagent (Millipore Sigma, cat# GERPN2235) and imaged using a BioRad VersaDoc Imaging system (BioRad) [19].

### 2.6. Peptide Docking

Peptide docking of LN to IL-1β (crystal structure, 9ilb) was determined using the CABS-dock web server (http://biocomp.chem.uw.edu.pl/CABSdock/ accessed on 28 January 2024). To create the model, the best prediction generated by CABS-dock was added to PyMOL 3.1  (Schrodinger, LLC, New York, NY, USA).

### 2.7. Immunoprecipitation

Immuno-precipitation (IP) of LN with IL-1β was performed using biotinylated LN peptides. Biotinylated LN [10 μg] and biotinylated SC were first incubated with Avidin-conjugated agarose beads (Thermo Fisher Scientific; cat# 20219) for 1 h at RT and washed thrice in PBS. Beads were then incubated with IL-1β [100 ng] for 30 min at RT, washed thrice in PBS, and boiled for 5 min in Laemmli buffer with DTT prior to Western blotting to identify IL-1β-LN interactions. Samples were electrophoresed via Western blotting on a 4–20% Tris-Glycine polyacrylamide gel and transferred to PVDF membrane. Blots were blocked in BSA and incubated with anti-LN antibody [1:500]. Blots were subsequently incubated with anti-mouse conjugated-HRP secondary antibody. Blots were washed and incubated with Amersham ECl Select Western blotting detection reagent and imaged using a BioRad VersaDoc MP 5000 Imaging system.

### 2.8. Competitive IP of IL1R1 with IL-1β

Protein A/G agarose beads (Thermo Fisher Scientific; cat# 20421) were first incubated with anti-His antibody. Following washing, anti-His bound Protein A/G agarose beads were incubated with IL1R1 [0.5 μg] and His-tagged IL-1β (IL-1β-His) [0.2 μg], alone or in combination with either unlabeled IL-1β [0.2 ng], LN [0.5 μg], or SC [0.5 μg] for 30 min. Beads were washed in PBS and processed for Western blotting via boiling for 5 min in Laemmli buffer with DTT. Lysates were electrophoresed on 4–20% gradient gels and transferred to 0.2 μM PVDF membranes. Blots were washed and probed with anti-IL1R1 antibody [1:1000], followed by goat anti-rabbit HRP-conjugated secondary antibody and detection with ECL Select. Blots were imaged using a BioRad VersaDoc MP 5000 Imaging system.

### 2.9. Ca^2+^-Mobilization

DRG neurons were isolated from the lumbar regions (L2–L5) of 12-week-old C57BL/6 mice, as described in Sleigh et al. [21]. Briefly, spinal columns were dissected and DRGs were excised, enzymatically digested, and cultured on glass chamber slides coated with poly-D-lysine. DRG neurons were seeded at a density of 10^5^ cells/cm^2^. To inhibit glial growth, cultures were treated with cytosine arabinoside [5 μM] for 24 h. After five days of culturing, DRGs were treated for seven days with IL-1β [5 ng/mL], with or without LN [1 μg/mL]. Cells were loaded with Fluo-4, AM, following the manufacturer’s instructions (ThermoFisher, Waltham, MA, USA; cat# F14217), and imaged for changes in intracellular Ca^2+^ either at resting state or following stimulation with capsaicin [100 nM] using a Zeiss LSM800 confocal microscope (Zeiss, Oberkochen, Germany).

### 2.10. Histology

IVD puncture model was performed in 4–6-month-old skeletally mature New Zealand White rabbits (Western Oregon Rabbit Co., Philomath, OR, USA) at the L2/3 and L4/5 levels by inserting an 18 G needle through the annulus fibrosus (AF) into the nucleus pulposus (NP) [17]. Two weeks following nucleotomy, discs were treated with intradiscal injections into the NP area with either the vehicle (saline) [10 μL/disc] or the LN peptide [25 μg/disc]. Twelve weeks post-treatment, rabbits were euthanized, and their discs were harvested for histological assessment. IVDs were fixed in 10% neutralized formalin, decalcified in Cal-Ex™ II Fixative/Decalcifier, and subsequently paraffin-embedded. Sagittal sections of 4 μm thickness were prepared from each IVD, dewaxed using Histo-Clear (Thermo Fisher Scientific, Waltham, MA, USA), and rehydrated through a graded ethanol series into distilled water. For immunohistochemical analysis, specimens were processed using Vectastain Elite ABC-HRP Peroxidase kit (Vector Laboratories, Newark, CA, USA; cat# PK-6100) and probed with anti-PGP9.5, anti-NGF, anti-CGRP, and anti-aggrecan antibodies. Following staining, sections were dehydrated and mounted using Permount™ (ThermoFisher Scientific, cat# SP15-100) for preservation. Images were captured using a Leica DM LB2 light microscope. An image analysis was performed using ImageJ version 1.54m Software.

### 2.11. Statistical Analysis

Data were analyzed using ANOVA followed by a Dunnett’s post hoc test. A *p*-value of less than 0.05 was considered statistically significant.

## 3. Results

### 3.1. LN Modulates IL-1β-Induced Gene Expression in hNP Cells

**Figure 1:**
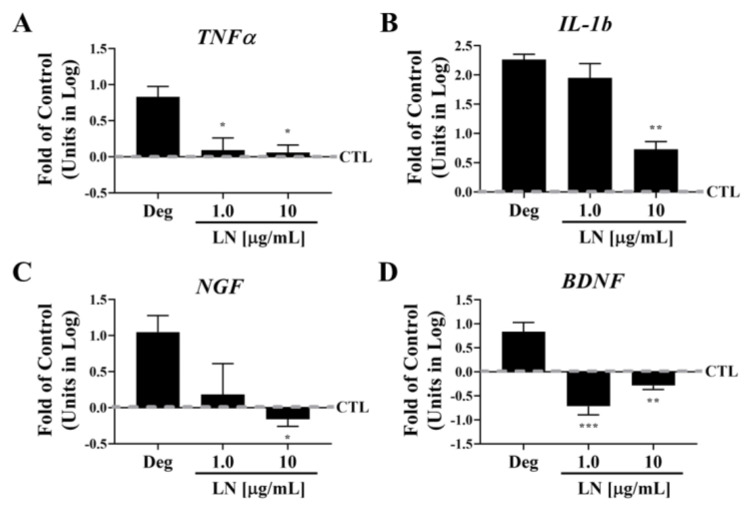
Effect of LN on IL-1β-induced gene expression in hNP cells. NP pellets were treated with IL-1β (Deg), LN [1 or 10 µg/mL] with IL-1β or PBS (CTL) for 6 days. Gene expression was measured by qPCR for (**A**) *TNF-α*, (**B**) *IL1-β*, (**C**) *NGF*, and (**D**) *BDNF* expression. Means ± SD; n = four donors; ANOVA, Dunnett’s post hoc multiple comparison test. ***, *p* < 0.001; **, *p* < 0.01; *, *p* < 0.05, comparison with control.

To assess the effects of LN on inflammatory and pain marker expression in IL-1β-stimulated hNP cells, NP cell pellets were treated with IL-1β (Deg), LN [1 or 10 µg/mL] with IL-1β, or PBS (control) for 6 days, and gene expression was measured via qPCR (Figure 1). The expression of the pro-inflammatory cytokine *TNFα* was significantly downregulated following either 1 or 10 µg/mL LN treatment (Figure 1A; *p* < 0.05). A similar trend was observed for *IL-1β* (Figure 1B). Two prominent pain factors regulated by IL-1β in hNP cells include *NGF* and *BDNF*. Treatment with LN downregulated both factors with either 1 or 10 µg/mL (Figure 1C,D). Although NP cells are the primary source of IL-1β, the AF is also a contributing factor [4,5]. To assess whether LN can regulate pain and inflammation markers in hAF cells, we co-incubated the cells with LN and IL-1β for six days, following the same protocol as with the treated hNP cells. As demonstrated in Figure S1, treatment with LN led to a dose-dependent downregulation of inflammatory markers *TNFα* and *IL-1β*, as well as pain markers *NGF* and *BDNF*.

### 3.2. LN Suppresses IL-1β-Induced NF-κB Activation in hNP Cells

**Figure 2:**
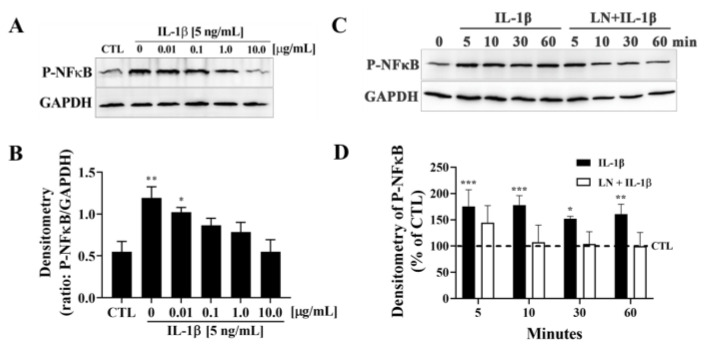
LN regulates IL-1β signalling in hNP cells. (**A**) Immunoblotting for P-NFκB following coincubation of IL-1β [5 ng/mL] and the indicated concentrations of LN for 10 min. Representative blots are presented. (**B**) Densitometry was performed on individual blots and normalized by GAPDH. Plots represent means ± SEMs. ANOVA Dunnett’s post hoc multiple comparisons test; * *p* < 0.05, ** *p* < 0.01; n = 4. (**C**) Western blot of P-NFκB in hNP cells following incubation with IL-1β [5 ng/mL], alone or in combination with LN [1 μg/mL], for the indicated times. GAPDH was blotted as a loading control. (**D**) Densitometry of blots presented in (**A**) demonstrating inhibition of P-NFκB by LN. ANOVA Dunnett’s post hoc multiple comparison test, CTL—0 min; *, *p* < 0.05; **, *p* < 0.01; ***, *p* < 0.001; n = 4. Original images of (**A**, **C**) can be found in Supplementary Materials.

To determine the effect of LN on IL-1β-induced NF-κB activation in hNP cells, phosphorylated NF-κB (P-NFκB) levels were assessed via immunoblotting. GAPDH was used as a loading control, and densitometric analysis was performed for quantification (Figure 2). IL-1β stimulation [5 ng/mL] resulted in a dose-dependent increase in P-NFκB expression compared to control cells (CTL) (Figure 2A,B). Notably, co-treatment with LN at concentrations of 1.0 and 10.0 µg/mL significantly reduced P-NFκB expression compared to IL-1β-treated cells alone (*p* < 0.05 and *p* < 0.01, respectively). Lower concentrations of LN did not show a significant inhibition.

The time-dependent effect of IL-1β on P-NFκB expression showed a peak at 10 min post-stimulation, which persisted for up to 60 min (Figure 2C). Co-treatment with LN [10 µg/mL] notably reduced P-NFκB expression at 30 and 60 min compared to IL-1β alone. Densitometric quantification confirmed a significant suppression of NF-κB activation by LN at these time points (*p* < 0.05, Figure 2D).

These findings demonstrate that LN inhibits IL-1β-induced NF-κB activation in hNP cells in both a dose- and time-dependent manner, highlighting its potential to suppress inflammation-associated signalling pathways in intervertebral disc degeneration.

### 3.3. Cytokine-Targeted Specificity of LN in hNP Cells

**Figure 3:**
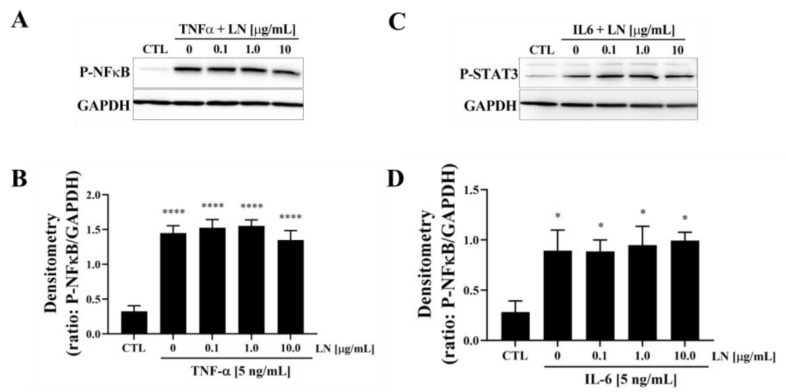
Effects of LN on pro-inflammatory cytokines. (**A**) TNF-α [5 ng/mL] or (**C**) IL-6 [5 ng/mL] for 10 min, followed by immunoblotting for P-NFκB. Representative blots are presented. (**B**,**D**) Densitometry was carried out on individual blots and normalized via GAPDH. Plots represent means ± SEMs. ANOVA Dunnett’s post hoc multiple comparisons test; * *p* < 0.05, **** *p* < 0.0001; n = 4. Original images of (**A**,**C**) can be found in Supplementary Materials.

To evaluate the cytokine-targeted specificity of LN, hNP cells were treated with TNFα or IL-6 in the presence of various concentrations of LN, and key downstream signalling pathways were analyzed using immunoblotting. Treatment with TNFα [10 ng/mL] significantly increased phosphorylated NF-κB (P-NFκB) levels compared to control cells (CTL) (Figure 3A). Co-treatment with LN at concentrations of 0.1, 1.0, and 10.0 µg/mL had no significant effect on TNFα-induced P-NFκB expression, as densitometric analysis showed persistently elevated levels across all LN concentrations (Figure 3B). This suggests that LN does not interfere with TNFα-mediated NF-κB activation. Similarly, IL-6 stimulation [5 ng/mL] led to a significant increase in phosphorylated STAT3 (P-STAT3) expression (Figure 3C). However, the co-treatment with LN had no significant effect on IL-6-induced P-STAT3 levels, as shown in the densitometric analysis (*p* > 0.05, Figure 3D).

### 3.4. LN Interacts Specifically with IL-1β: Peptide Docking and Immunoprecipitation

**Figure 4:**
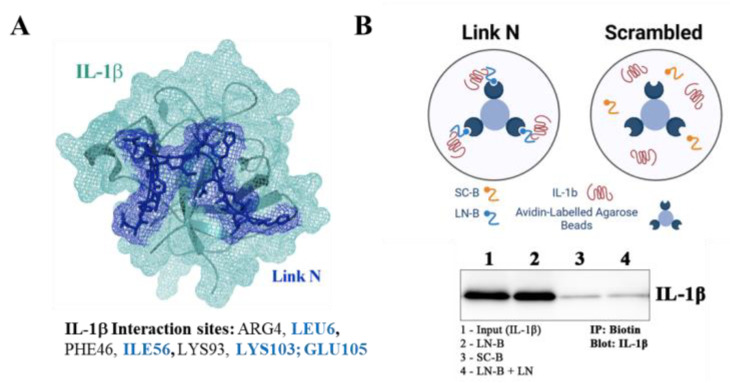
Peptide docking and immunoprecipitation of LN with IL-1β. (**A**) Peptide docking of LN to IL-1β (crystal structure, 9ilb) was determined using CABS-dock web server. Model was created using PyMOL (Schrodinger, LLC). IL-1β residues known to be important in structure–function activity with the IL-1β type 1 receptor are listed, and those predicted to interact with LN based on simulation are highlighted in blue. (**B**) Immuno-precipitation (IP) of LN with IL-1β. Biotinylated LN or biotinylated scrambled LN (SC) was attached to Avidin-labelled agarose beads and then incubated with IL-1β. Western blotting was performed to identify IL-1β-LN interactions. Lane 1: CTL (PBS) with IL-1β; lane 2: IP of LN with IL-1β; lane 3: SC with IL-1β; lane 4: IL-1β with LN-B + free LN. Original images of (**B**) can be found in Supplementary Materials.

To investigate the molecular interaction between LN and IL-1β, peptide docking simulations and immunoprecipitation assays were performed. A docking analysis using the CABS-dock web server revealed the predicted interaction sites of LN with IL-1β (Figure 4A). Critical IL-1β residues known to mediate interaction with its type 1 receptor include LEU6, ILE56, LYS103, and GLU109. Residues predicted to interact with LN based on simulation are highlighted in blue, suggesting that LN binds IL-1β at functionally important regions, potentially interfering with IL-1β receptor interactions.

The interaction between LN and IL-1β was further validated using immunoprecipitation (Figure 4B). Biotinylated LN was attached to avidin-labelled agarose beads and incubated with IL-1β. Western blot analysis demonstrated a clear interaction between LN and IL-1β (Lane 2), while scrambled LN (SC) did not bind to IL-1β (Lane 3). IL-1β alone (Lane 4) and the control (PBS with IL-1β, Lane 1) served as additional references.

These findings confirm that LN interacts specifically with IL-1β at residues that are important for its receptor-binding function. This interaction likely underlies the ability of LN to modulate IL-1β signalling, providing a mechanistic basis for its anti-inflammatory effects.

### 3.5. LN Competitively Inhibits IL-1β Binding to IL-1 Receptor Type 1

**Figure 5:**
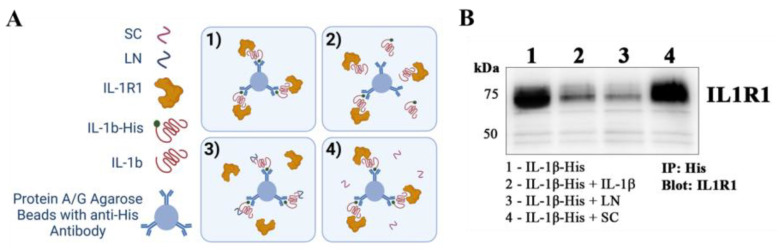
Competitive immunoprecipitation of IL1R1 with IL-1β and LN. (**A**) Schematic of the immuno-precipitation (IP) of IL1R1 with His-tagged IL-1β (IL-1β-His) in the absence or presence of LN, SC, or IL-1β. (**B**) Western blotting was performed to identify IL-1β-IL1R1 interactions. Lane 1: CTL with His-IL-1β; lane 2: His-IL-1β and IL-1β [1:1]; lane 3: IL-1β-His and LN; lane 4: IL-1β-His and scrambled LN (SC). Original images of (**B**) can be found in Supplementary Materials.

To determine whether LN interferes with the binding of IL-1β to its receptor IL1R1, competitive immunoprecipitation assays were performed using His-tagged IL-1β (IL-1β-His) in the presence or absence of LN, scrambled peptide (SC), or unlabeled IL-1β. A schematic of the immunoprecipitation protocol is presented in Figure 5A.

Western blot analysis was used to detect the IL-1β-IL1R1 interactions (Figure 5B). In the control lane (Lane 1), IL-1β-His showed a baseline interaction with IL1R1. When IL-1β-His was incubated with an equimolar amount of unlabeled IL-1β (1:1), a significant reduction in IL1R1 binding was observed (Lane 2), indicating successful competition by unlabeled IL-1β. Notably, the addition of LN to IL-1β-His (Lane 3) resulted in a marked decrease in IL1R1 detection compared to the control, suggesting that LN competes with IL-1β for binding to IL1R1. In contrast, the scrambled peptide (SC) did not affect the IL-1β-His and IL1R1 interaction (Lane 4), as evidenced by IL1R1 levels, which were similar to the control.

These results demonstrate that LN can competitively inhibit the binding of IL-1β to IL1R1, likely by directly interacting with IL-1β and preventing its engagement with the receptor. This competitive inhibition provides a potential mechanism for the anti-inflammatory effects of LN, as it may disrupt the IL-1β-mediated signalling pathways involved in intervertebral disc degeneration.

### 3.6. LN Modulates IL-1β-Induced DRG Hypersensitivity

**Figure 6:**
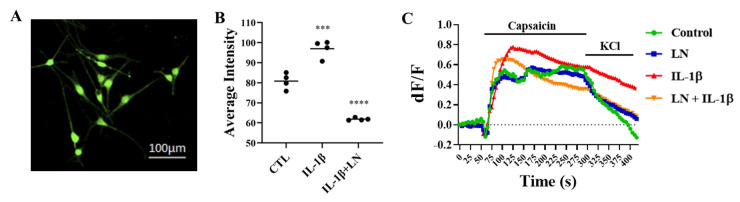
LN modulates IL-1β-induced DRG hypersensitivity. Isolated DRG neurons were treated with IL-1β or IL-1β and LN for 7 days. (**A**) Confocal image showing DRG neurons loaded with Fluo-4 calcium indicator. (**B**) Individual plot of the averaged basal intensities of neurons following IL-1β with or without LN. (**C**) Calcium imaging data of DRG neurons exposed to capsaicin, followed by the addition of KCl. ANOVA, posthoc Dunnett’s multiple comparison test; ***, *p* < 0.001, ****, *p* < 0.0001; n = 4 (25 cells per experiment).

DRGs were isolated from murine lumbar spines and cultured on chamber slides. DRGs were incubated with IL-1β, alone or in combination with LN for 7 days, then loaded with a Ca^2+^ indicator to measure changes in intracellular Ca^2+^-flux (Figure 6A). The relative basal Ca^2+^ levels were elevated following IL-1β treatment; however, in the presence of LN, this effect was mitigated (Figure 6B). When DRGs were exposed to capsaicin to activate Ca^2+^ influx, Ca-imaging indicated that DRGs pre-treated with IL-1β showed a heightened response that was refractory when the stimulus was removed. This response reversed with the co-incubation of IL-1β and LN (Figure 6C).

### 3.7. LN Reduces Pain-Related Markers in a Rabbit Model of Disc Degeneration

**Figure 7:**
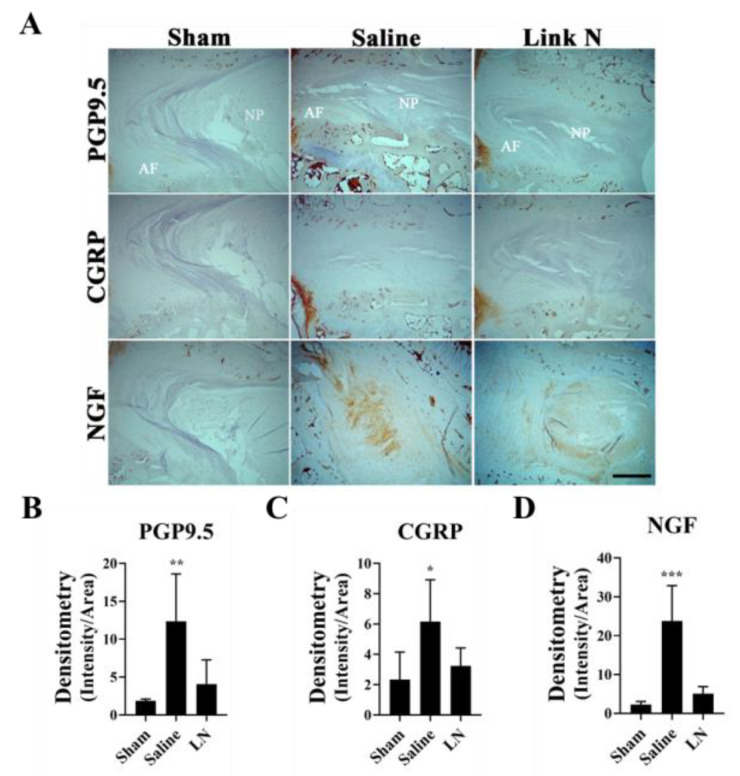
LN modulates markers of pain in a rabbit model of disc degeneration. Rabbit discs were subjected to a puncture model of disc degeneration followed by a single injection of saline or LN. Twelve weeks post-injection, discs were measured for markers of pain in disc tissue. (**A**) IHC demonstrating expression of PGP9.5, CGRP, and NGF. (**B**–**D**) are densitometric graphs of the expression of PGP9.5, CGRP, and NGF of the images presented in (**A**). Means ± SD; n = four rabbits. ANOVA, posthoc Dunnett’s multiple comparison test. ***, *p* < 0.001; **, *p* < 0.01; *, *p* < 0.05, comparison with sham discs. Scale bar = 100 μm.

To investigate the effects of LN treatment on markers of pain in a rabbit model of disc degeneration, immunohistochemical (IHC) analysis was performed to examine the expression of PGP9.5, CGRP, and NGF in disc tissue 12 weeks post-injection. Representative images of these markers in Sham, Saline-treated, and LN-treated discs are shown in Figure 7A. The neuronal marker PGP9.5 was highly expressed in the AF region of saline-treated discs, as evidenced by intense staining (Figure 7A). In contrast, LN treatment significantly reduced PGP9.5 staining compared to the saline group, with levels approaching those seen in sham discs. The quantification of PGP9.5 expression revealed a significant increase in the saline group compared to sham (*p* < 0.001), while LN treatment significantly reduced PGP9.5 expression (*p* < 0.01, Figure 7B). Similar results were observed for the expression of CGRP, a marker of sensory nerve fibres (Figure 7A). LN treatment appeared to reduce CGRP expression relative to Saline-treated discs, although not to the same levels as Sham discs. Densitometric analysis demonstrated a significant increase in CGRP expression in saline-treated discs compared to sham (*p* < 0.05), whereas LN treatment resulted in a non-significant reduction in CGRP expression compared to the saline group (Figure 7C). Finally, a key mediator of pain and nerve growth, NGF, was strongly expressed in saline-treated discs, but significantly reduced in LN-treated discs (Figure 7A,D; *p* < 0.001). LN treatment markedly reduced NGF staining compared to Saline, with levels similar to those observed in Sham discs. Quantitative analysis confirmed a significant increase in NGF expression in Saline-treated discs compared to Sham (*p* < 0.001), while LN treatment significantly reduced NGF levels (*p* < 0.01, Figure 7D).

One characteristic of LN initially described in the disc is the ability to induce repair in disc models. This was demonstrated through its upregulation of matrix proteins such as proteoglycans and collagen [17,22]. Figure S2A shows that LN dose-dependently increased the expression of the matrix protein aggrecan (*ACAN*) when co-incubated with IL-1β in human NP cells. To determine whether LN can also increase the synthesis of aggrecan in IVDs, we performed IHC on discs from the rabbit disc puncture model (Figure S2B). In the saline-treated IVDs, aggrecan synthesis was largely reduced; however, when LN was administered, aggrecan appeared to mimic the IVDs in sham-treated animals (Figure S2B).

### 3.8. Summary of LN Effects on NP Cellular Activity

**Figure 8:**
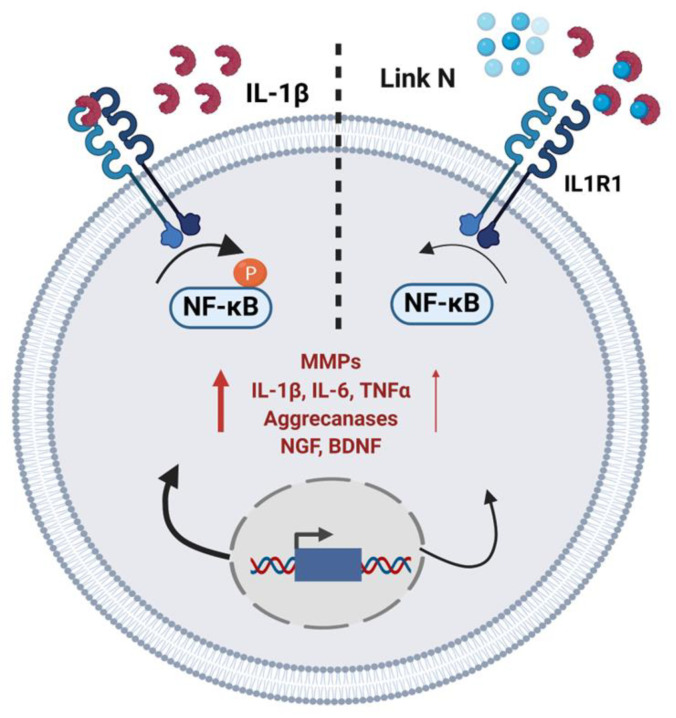
Schematic summarizing the effects of LN on NP cellular activity.

Figure 8 provides a schematic representation of the dual effects of LN on NP cells, highlighting its anti-inflammatory properties.

## 4. Discussion

IVD degeneration is a leading cause of chronic low back pain, with inflammation and ECM breakdown playing central roles in disease progression [4]. In this study, we demonstrate that LN, a small peptide derived from the N-terminus of the link protein with anabolic-like effects [23,24], also exhibits anti-inflammatory properties in NP cells. Our findings demonstrate that LN directly interacts with IL-1β, effectively inhibiting its signalling and modulating its downstream effects in NP cells and DRG neurons, offering a potential therapeutic strategy for mitigating inflammation-associated disc degeneration.

Inflammation is a critical driver of disc degeneration, characterized by elevated levels of pro-inflammatory cytokines such as IL-1β, TNFα, and IL-6, which exacerbate ECM degradation through the upregulation of matrix-degrading enzymes (MMPs, aggrecanases) [2,23,25]. Our findings indicate that LN specifically targets IL-1β signalling, inhibiting NF-κB phosphorylation and downstream inflammatory mediators (Figure 2). Immunoprecipitation and peptide docking studies further demonstrate that LN competitively inhibits IL-1β binding to its receptor, IL1R1, thereby preventing receptor activation and downstream signalling (Figure 4 and Figure 5). In contrast, LN does not significantly interfere with TNFα- or IL-6-mediated signalling, as evidenced by the sustained NF-κB and STAT3 activation (Figure 3). This specificity is an important finding, as it suggests that the effects of LN are selective for IL-1β, known as a key mediator in disc degeneration pathogenesis [2]. Given that IL-1β levels are elevated in degenerative discs, targeting this pathway may provide a more focused therapeutic approach [26,27]. Although LN was not observed to alter the activation of NF-κB and STAT3 by TNFα and IL-6, respectively, other downstream factors may be altered. Future studies can include an investigation of apoptotic and senescence pathways. 

The peptide docking simulations and immunoprecipitation assays provide further insight into how LN interferes with IL-1β activity (Figure 4 and Figure 5). LN interacts with residues of IL-1β that are known to be important for receptor binding, such as LEU6, ILE56, and GLU109. By occupying these sites, LN likely prevents IL-1β from engaging IL1R1, thereby blocking receptor activation and NF-κB signalling. This competitive inhibition represents a mechanism of action distinct from the traditional anti-inflammatory therapies that target downstream signalling components [28].

In addition to its anti-inflammatory effects, LN also modulates the neuroinflammatory pathways associated with pain in IVD degeneration. IL-1β has been shown to enhance nociceptive signalling by promoting the expression of neurotrophic factors such as NGF and BDNF to stimulate sensory nerve growth [3,7,11]. Our findings indicate that LN treatment in a rabbit model of IVD degeneration attenuates the expression of pain-related markers, including PGP9.5, CGRP, and NGF, in degenerative discs, suggesting its potential to reduce nociceptive sensitization.

Furthermore, pain modulation is a complex process involving neuroplasticity, which plays a crucial role in chronic pain. The results of our study suggest that LN can modulate IL-1β-induced neuronal activity. The elevated intracellular Ca^2+^ levels observed in IL-1β-treated DRG neurons indicate increased neuronal excitability, which aligns with previous findings on IL-1β-induced hyperalgesia [29]. The ability of LN to significantly reduce Ca^2+^ levels and restore desensitization upon capsaicin stimulation supports its potential as a neuroprotective agent against IL-1β-induced inflammatory pain conditions. These findings highlight the importance of modulating pro-inflammatory cytokine activity to potentially control sensory neuron hypersensitivity and chronic pain development. The limitation in our DRG experiment was the use of monocultures. Since NP cells are the principal cells that secrete IL-1β, future studies with LN will include co-cultures of NP and DRGs.

While inflammation drives tissue degradation, the loss of ECM components such as aggrecan and type II collagen further accelerates disc degeneration [23,24]. LN has been shown to activate BMP signaling, a key anabolic pathway involved in maintaining matrix homeostasis in NP cells [30,31]. Specifically, LN promotes the phosphorylation of Smad1/5 downstream of BMPR1/2, leading to the increased expression of anabolic genes, including COL2A1 and ACAN [30,31]. The ability of LN to activate this pathway provides mechanistic insight into its regenerative potential [16,30]. By promoting the synthesis of aggrecan and collagen [19], LN offers a regenerative approach to degenerative discs and simultaneously prevents further degradation through its anti-inflammatory effects, as demonstrated in the current study.

Unlike conventional treatments that primarily focus on pain management, LN directly targets the underlying pathology of IVD degeneration [13]. Our findings demonstrate that LN interacts with IL-1β, regulating its interaction with IL1R1. This specificity for IL-1β further underscores its potential as a targeted anti-inflammatory agent, reducing IL-1β-driven inflammation. By inhibiting IL-1β-mediated pro-inflammatory signalling and enhancing anabolic-like properties in disc cells, LN provides a dual approach to supressing inflammation while promoting ECM synthesis in IVD degeneration [16,17].

While our study provides strong mechanistic evidence for the therapeutic potential of LN, it is not without limitations. First, the study primarily relies on in vitro models of NP cells, which may not fully capture the complexity of the IVD microenvironment. Second, while LN selectively inhibits IL-1β, further studies are needed to determine its effects in the presence of multiple cytokines, as seen in degenerative discs. Finally, in vivo studies will be needed to validate the modulation of IL-1β-induced DRG hypersensitivity and explore any potential behavioural outcomes following LN treatment.

Despite these limitations, our findings provide the basis for potential clinical applications. LN could be administered via targeted intradiscal injections, providing direct local treatment and minimizing systemic exposure. Controlled-release formulations, including hydrogel or nanoparticle delivery systems, might further enhance therapeutic outcomes by ensuring sustained peptide availability within the disc environment. Additionally, LN could potentially be delivered in combination therapies involving other regenerative agents, such as mesenchymal stem cells, to synergistically enhance its regenerative capacity. These minimally invasive and targeted strategies not only maximize the therapeutic potential of LN but also facilitate clinical adoption for treating disc degeneration and chronic back pain.

## 5. Conclusions

This study demonstrates a mechanism for the anti-inflammatory effects of LN. By targeting both inflammatory and degenerative pathways, LN holds significant promise for addressing the multifactorial nature of disc degeneration and restoring tissue homeostasis.

## Figures and Tables

**Table 1:** Primer sequences for human genes.

Human Genes	Primer Sequence	Size (bp)
*h-IL1β*	F (390–410): 5′-ACCTATCTTCTTCGACACATG-3′R (537–557): 5′-ACCACTTGTTGCTCCATATCC-3′	148
*h-TNFα*	F (310–326): 5′-ACC ACG CTC TTC TGC CT-3′R (436–456): 5′-TAC AAC ATG GGC TAC AGG CTT-3′	127
*h-NGF*	F (302–321): 5′-TCA GCA TTC CCT TGA CAC TG-3′R (521–540): 5′-TGC TCC TGT GAG TCC TGT TG-3′	220
*h-BDNF*	F (481–501):5′-GAC ATC ATT GGC TGA CAC TTT-3′R (586–605): 5′-TAC TGA GCA TCA CCC TGG AC-3′	106
*h-GAPDH*	F (113–133): 5′-TGA AGG TCG GAG TCA ACG GAT-3′R (273–293): 5′-TTC TCA GCC TTG ACG GTG CCA-3′	181

## Data Availability

All the authors are affiliated with Antoniou and Mwale’s Lab at the Lady Davis Institute, where the research was conducted. The data were shared internally via the institute’s network, and all authors had full access throughout the study. The original contributions presented in the study are included in the article/Supplementary Material, further inquiries can be directed to the corresponding author.

## Supplementary Materials

The following supporting information can be downloaded at: https://www.mdpi.com/article/10.3390/biom15040603/s1, Figure S1. Effect of LN on IL-1β-induced gene expression in human AF cells. Figure S2. Anabolic effects of LN on aggrecan expression. Figure S3. LN regulates IL-1β signaling in hNP cells. Figure S4. LN regulates IL-1β signaling in hNP cells.  Figure S5. Effects of LN on pro-inflammatory cytokines. Figure S6. Effects of LN on pro-inflammatory cytokines. Figure S7. Immunoprecipitation of LN with IL-1β. Figure S8. Competitive Immunoprecipitation of IL1R1 with IL-1β and LN.

## Abbreviations

The following abbreviations are used in this manuscript:

IVD	Intervertebral Disc
NP	Nucleus Pulposus
AF	Annulus Fibrosis
IP	Immunoprecipitation
IHC	Immunohistochemistry
LN	Link N
DRG	Dorsal Root Ganglion
NGF	Nerve Growth Factor

## References

[1] Sliwa K., Global Burden of Disease Study 2013 Collaborators (2015). Lancet, Global, regional, and national incidence, prevalence, and years lived with disability for 301 acute and chronic diseases and injuries in 188 countries, 1990–2013: A systematic analysis for the Global Burden of Disease Study 2013. Lancet.

[2] Risbud M.V., Shapiro I.M. (2014). Role of cytokines in intervertebral disc degeneration: Pain and disc content. Nat. Rev. Rheumatol..

[3] Emanuel K.S., Mader K., Peeters M., Kingma I., Rustenburg C., Vergroesen P.-P., Sammon C., Smit T. (2018). Early changes in the extracellular matrix of the degenerating intervertebral disc, assessed by Fourier transform infrared imaging. Osteoarthr. Cartil..

[4] Diwan A.D., Melrose J. (2023). Intervertebral disc degeneration and how it leads to low back pain. JOR Spine.

[5] Lyu F.-J., Cui H., Pan H., MC Cheung K., Cao X., Iatridis J.C., Zheng Z. (2021). Painful intervertebral disc degeneration and inflammation: From laboratory evidence to clinical interventions. Bone Res..

[6] Freemont A.J. (2009). The cellular pathobiology of the degenerate intervertebral disc and discogenic back pain. Rheumatology.

[7] Meisel H.J., Agarwal N., Hsieh P.C., Skelly A., Park J.-B., Brodke D., Wang J.C., Yoon S.T., Buser Z. (2019). Cell Therapy for Treatment of Intervertebral Disc Degeneration: A Systematic Review. Glob. Spine J..

[8] Richardson S.M., Doyle P., Minogue B.M., Gnanalingham K., A Hoyland J. (2009). Increased expression of matrix metalloproteinase-10, nerve growth factor and substance P in the painful degenerate intervertebral disc. Arthritis Res. Ther..

[9] La Binch A., Cole A.A., Breakwell L.M., Michael A.L., Chiverton N., Cross A.K., Le Maitre C.L. (2014). Expression and regulation of neurotrophic and angiogenic factors during human intervertebral disc degeneration. Arthritis Res. Ther..

[10] Keefe K.M., Sheikh I.S., Smith G.M. (2017). Targeting Neurotrophins to Specific Populations of Neurons: NGF, BDNF, and NT-3 and Their Relevance for Treatment of Spinal Cord Injury. Int. J. Mol. Sci..

[11] García-Cosamalón J., Del Valle M.E., Calavia M.G., García-Suárez O., López-Muñiz A., Otero J., Vega J.A. (2010). Intervertebral disc, sensory nerves and neurotrophins: Who is who in discogenic pain?. J. Anat..

[12] Raj P.P. (2008). Intervertebral disc: Anatomy-physiology-pathophysiology-treatment. Pain Pract..

[13] Miller R.J., Jung H., Bhangoo S.K., White F.A. (2009). Cytokine and chemokine regulation of sensory neuron function. Sens. Nerves.

[14] Miller R.E., Miller R.J., Malfait A.M. (2014). Osteoarthritis joint pain: The cytokine connection. Cytokine.

[15] Knezevic N.N., Mandalia S., Raasch J., Knezevic I., Candido K.D. (2017). Treatment of chronic low back pain-new approaches on the horizon. J. Pain Res..

[16] Wang Z., Weitzmann M.N., Sangadala S., Hutton W.C., Yoon S.T. (2013). Link Protein N-terminal Peptide Binds to *Bone Morphogenetic Protein* (BMP) Type II Receptor and *Drives Matrix Protein* Expression in Rabbit Intervertebral Disc Cells*. J. Biol. Chem..

[17] Mwale F., Masuda K., Grant M.P., Epure L.M., Kato K., Miyazaki S., Cheng K., Yamada J., Bae W.C., Muehleman C. (2018). Short Link N promotes disc repair in a rabbit model of disc degeneration. Arthritis Res. Ther..

[18] Alaqeel M., Grant M., Epure L., Salem O., AlShaer A., Huk O., Bergeron S., Zukor D., Kc R., Im H.-J. (2020). Link N suppresses interleukin-1beta-induced biological effects on human osteoarthritic cartilage. Eur. Cell Mater..

[19] Noorwali H., Grant M.P., Epure L.M., Madiraju P., Sampen H., Antoniou J., Mwale F. (2018). Link N as a therapeutic agent for discogenic pain. JOR Spine.

[20] Livak K.J., Schmittgen T.D. (2001). Analysis of relative gene expression data using real-time quantitative PCR and the 2(-Delta Delta C(T)) Method. Methods.

[21] Sleigh J.N., West S.J., Schiavo G. (2020). A video protocol for rapid dissection of mouse dorsal root ganglia from defined spinal levels. BMC Res. Notes.

[22] Bach F.C., Laagland L.T., Grant M.P., Creemers L.B., Ito K., Meij B.P., Mwale F., Tryfonidou M.A. (2017). Link-N: The missing link towards intervertebral disc repair is species-specific. PLoS ONE.

[23] Wuertz K., Haglund L. (2013). Inflammatory mediators in intervertebral disk degeneration and discogenic pain. Glob. Spine J..

[24] Molinos M., Almeida C.R., Caldeira J., Cunha C., Gonçalves R.M., Barbosa M.A. (2015). Inflammation in intervertebral disc degeneration and regeneration. J. R. Soc. Interface.

[25] Le Maitre C.L., Hoyland J.A., Freemont A.J. (2007). Catabolic cytokine expression in degenerate and herniated human intervertebral discs: IL-1beta and TNFalpha expression profile. Arthritis Res. Ther..

[26] Pezet S., McMahon S.B. (2006). Neurotrophins: Mediators and modulators of pain. Annu. Rev. Neurosci..

[27] Burke J.G., Watson R.W.G., McCormack D., Dowling F.E., Walsh M.G., Fitzpatrick J.M. (2002). Intervertebral discs which cause low back pain secrete high levels of proinflammatory mediators. J. Bone Jt. Surg. Br..

[28] Kamali A., Ziadlou R., Lang G., Pfannkuche J., Cui S., Li Z., Richards R.G., Alini M., Grad S. (2021). Small molecule-based treatment approaches for intervertebral disc degeneration: Current options and future directions. Theranostics.

[29] Fujita D., Matsuoka Y., Yamakita S., Horii Y., Ishikawa D., Kushimoto K., Amino H., Amaya F. (2024). Rapid cleavage of IL-1β in DRG neurons produces tissue injury-induced pain hypersensitivity. Mol. Pain.

[30] Wang Z., Hutton W.C., Yoon S.T. (2013). ISSLS Prize winner: Effect of link protein peptide on human intervertebral disc cells. Spine.

[31] Bobick B.E., Chen F.H., Le A.M., Tuan R.S. (2009). Regulation of the chondrogenic phenotype in culture. Birth Defects Res. C Embryo Today.

