# Supplementary material for: Link N Directly Targets IL-1β to Suppress Inflammation and Regulate Sensory Pain in Intervertebral Disc Degeneration
Source: Biomolecules. 2025 Apr 19;15(4):603. doi: 10.3390/biom15040603 (PMC12024905; doi:10.3390/biom15040603)
Supplement: Supplementary file 1 [file biomolecules-15-00603-s001.zip › biomolecules-3547674-original image-Western Blot.pdf]

Supplementary Data

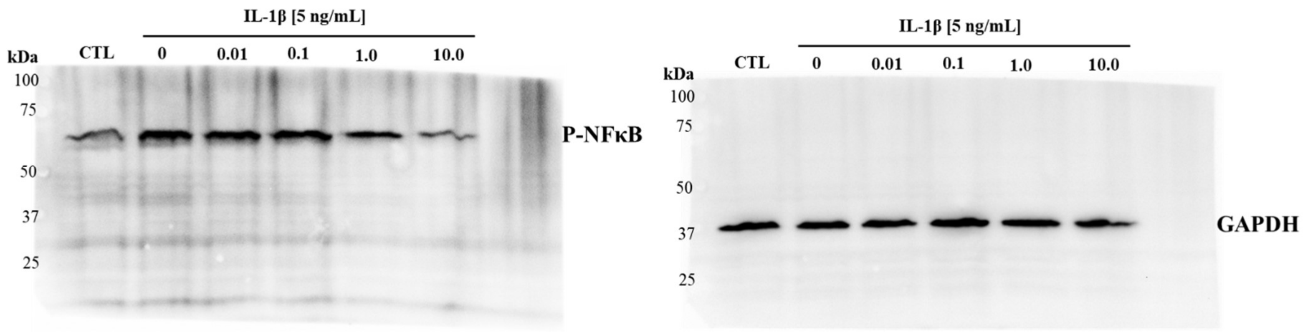

Figure S3. LN regulates IL-1β signaling in hNP cells. Immunoblotting for P-NFκB following coincubation of IL-1β [5 ng/mL] and the indicated concentrations of LN for 10 min. Representative blots are presented.

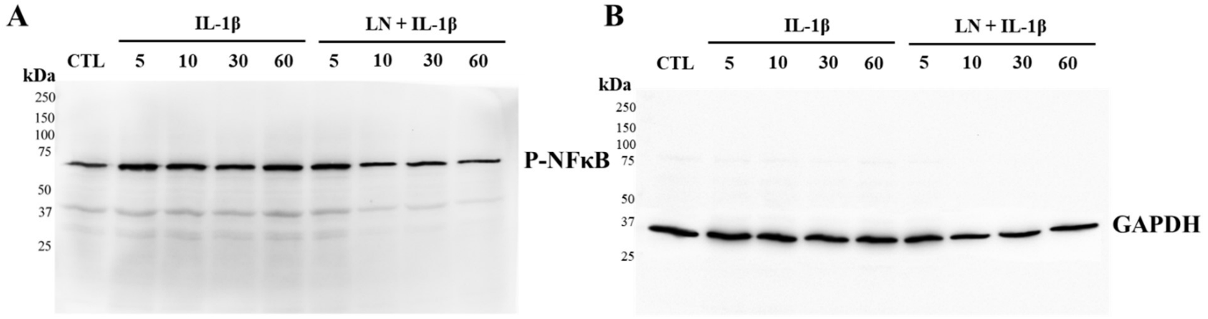

Figure S4. LN regulates IL-1β signaling in hNP cells. (A) Immunoblotting for P-NFκB following coincubation of IL-1β [5 ng/mL] and the indicated concentrations of LN for 10 min. Representative blots are presented. **(B)** Western blot of P-NFκB in hNP cells following incubation with IL-1β [5 ng/mL] alone or in combination with LN [1 μg/mL] for the indicated times. GAPDH was blotted as a loading control.

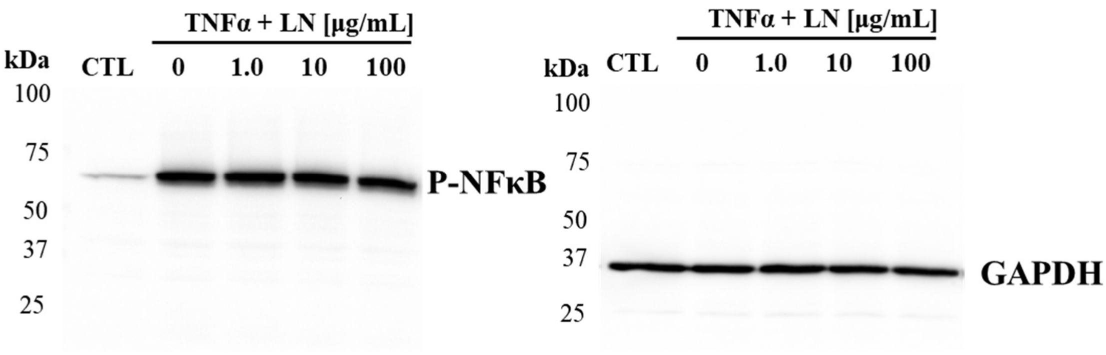

Figure S5. Effects of LN on pro-inflammatory cytokines. TNF-α [5 ng/mL] in combination with the indicated concentrations of LN for 10 min followed by immunoblotting for P-NFκB. Representative blots are presented.

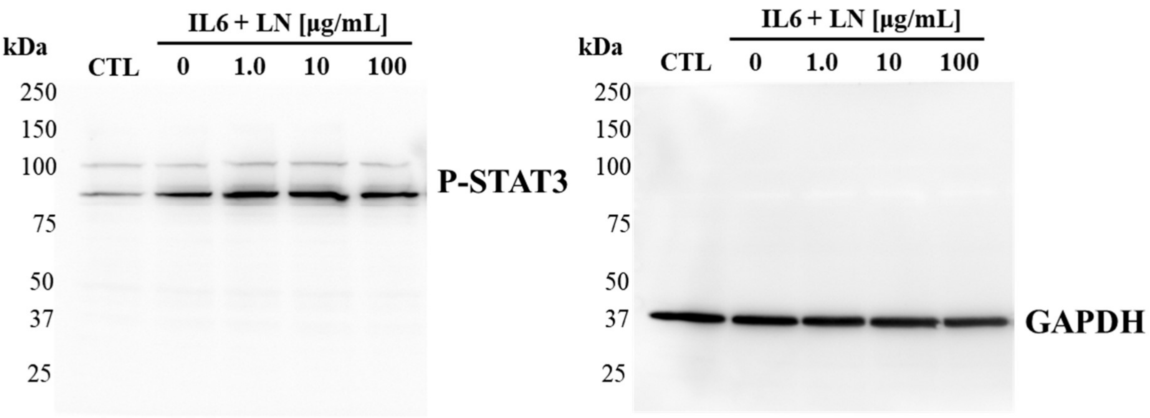

Figure S6. Effects of LN on pro-inflammatory cytokines. IL6 [10 ng/mL] in combination with the indicated concentrations of LN for 10 min followed by immunoblotting for P-NFκB. Representative blots are presented.

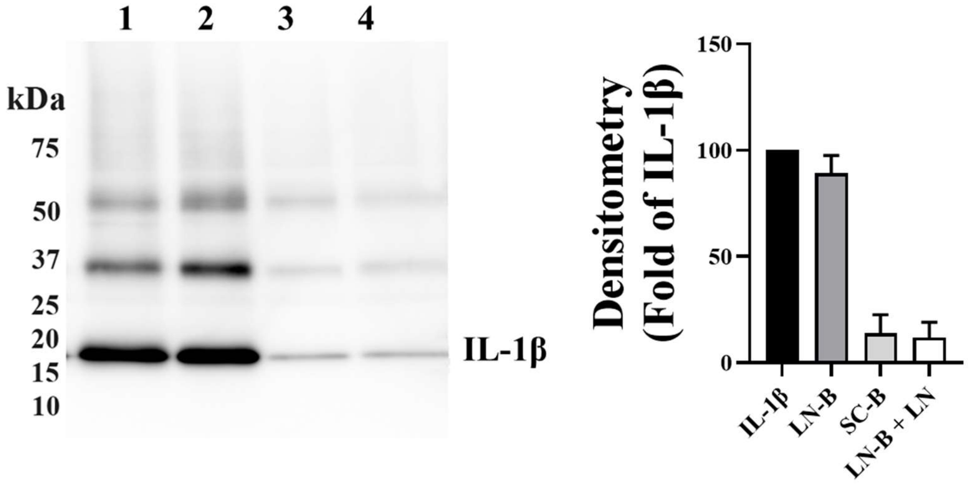

Figure S7. Immunoprecipitation of LN with IL-1β. (A) Immuno-precipitation (IP) of LN with IL-1β. Biotinylated LN or biotinylated scrambled LN (SC) was attached to Avidin-labelled agarose beads and then incubated with IL-1β. Western blotting was performed to identify IL-1β-LN interaction. Lane 1: IL-1β (input); lane 2: IP of LN with IL-1β; lane 3: IP of SC with IL-1β; lane 4: IP of LN-B plus free LN. **(B)** Densitometry of blots presented in (A); n=3.

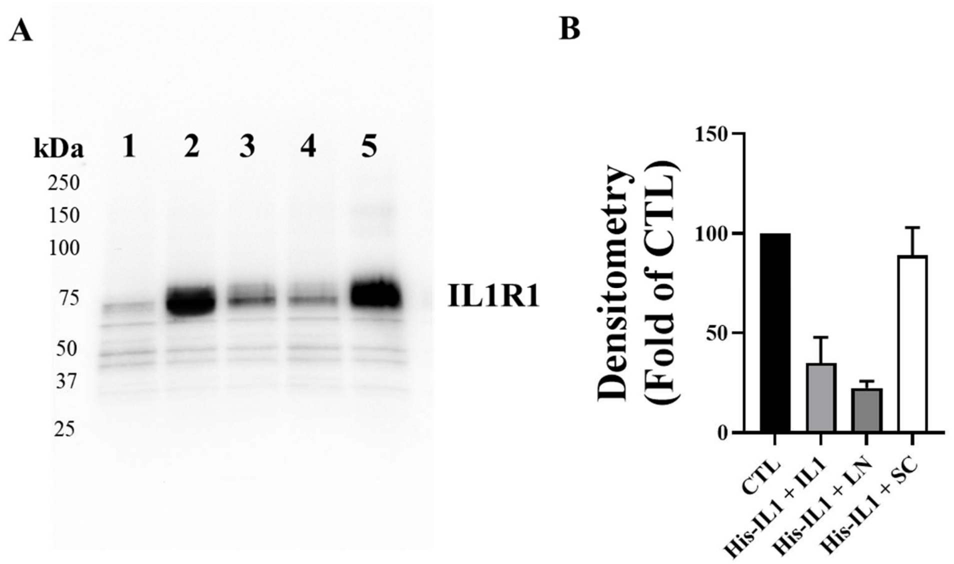

Figure S8. Competitive Immunoprecipitation of IL1R1 with IL-1β and LN. (A) Western blot demonstrating IL-1β-IL1R1 interactions. Lane 1: Agarose beads and IL1R1; lane 2: CTL with His-IL-1β; lane 3: His-IL-1β and IL-1β [1:1]; lane 4: IL-1β-His and LN; lane 5: IL-1β-His and scrambled LN (SC). **(B)** Densitometry of blots presented in (A); n=3.
