# Supplementary material for: Link N Directly Targets IL-1β to Suppress Inflammation and Regulate Sensory Pain in Intervertebral Disc Degeneration
Source: Biomolecules. 2025 Apr 19;15(4):603. doi: 10.3390/biom15040603 (PMC12024905; doi:10.3390/biom15040603)
Supplement: Supplementary file 1 [file biomolecules-15-00603-s001.zip › biomolecules-3547674-supplementary.pdf]

Supplementary Materials:

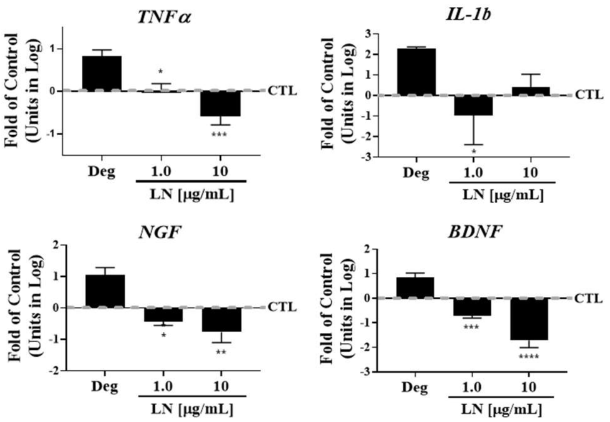

Figure S1. Effect of LN on IL-1β-induced gene expression in human AF cells. AF pellets were treated with IL-1β (Deg), LN [1 or 10 μg/mL] with IL-1β or PBS (CTL) for 6 days. Gene expression was measured by qPCR. Means ± SD; n = 4 donors; ANOVA, posthoc Dunnett's multiple comparison test. ***, $p < 0.001$; **, $p < 0.01$; *, $p < 0.05$, comparison with control; #, $p < 0.05$, comparison with IL-1β.

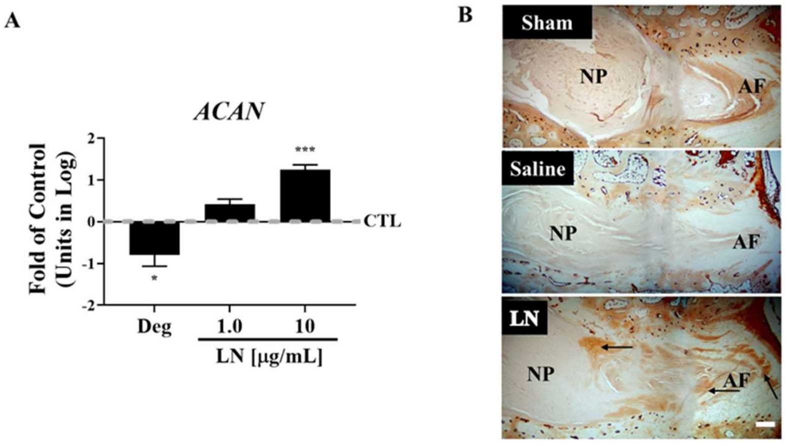

Figure S2. Anabolic effects of LN on aggrecan expression. A) NP pellets were treated with IL-1β (Deg), LN [1 or 10 μg/mL] with IL-1β or PBS (CTL) for 6 days. Gene expression was measured by qPCR. Means ± SD; n = 4 donors; ANOVA, posthoc Dunnett's multiple comparison test. ***, $p < 0.001$; *, $p < 0.05$, comparison with control. B) Rabbit discs were subjected to a puncture model of disc degeneration followed by a single injection of saline or LN. Twelve weeks post injection discs were imaged for aggrecan synthesis in disc tissue. Black arrows show aggrecan synthesis following LN treatment. Scale bar = 100 μm.
