# Supplementary figures and images for: Link N Directly Targets IL-1β to Suppress Inflammation and Regulate Sensory Pain in Intervertebral Disc Degeneration
Source: Biomolecules. 2025 Apr 19;15(4):603. doi: 10.3390/biom15040603 (PMC12024905; doi:10.3390/biom15040603)

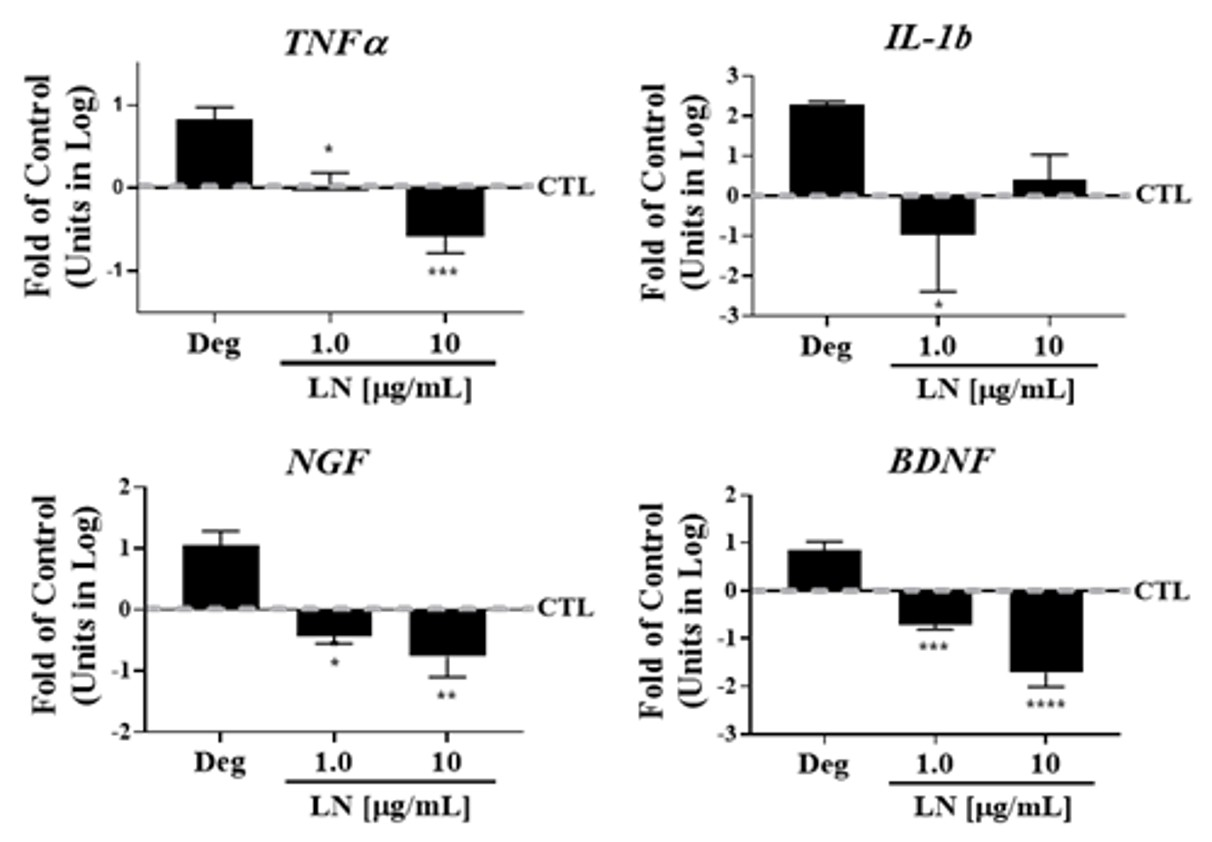

Supplement: Supplementary file 1 [file biomolecules-15-00603-s001.zip › Figure S1.jpg]

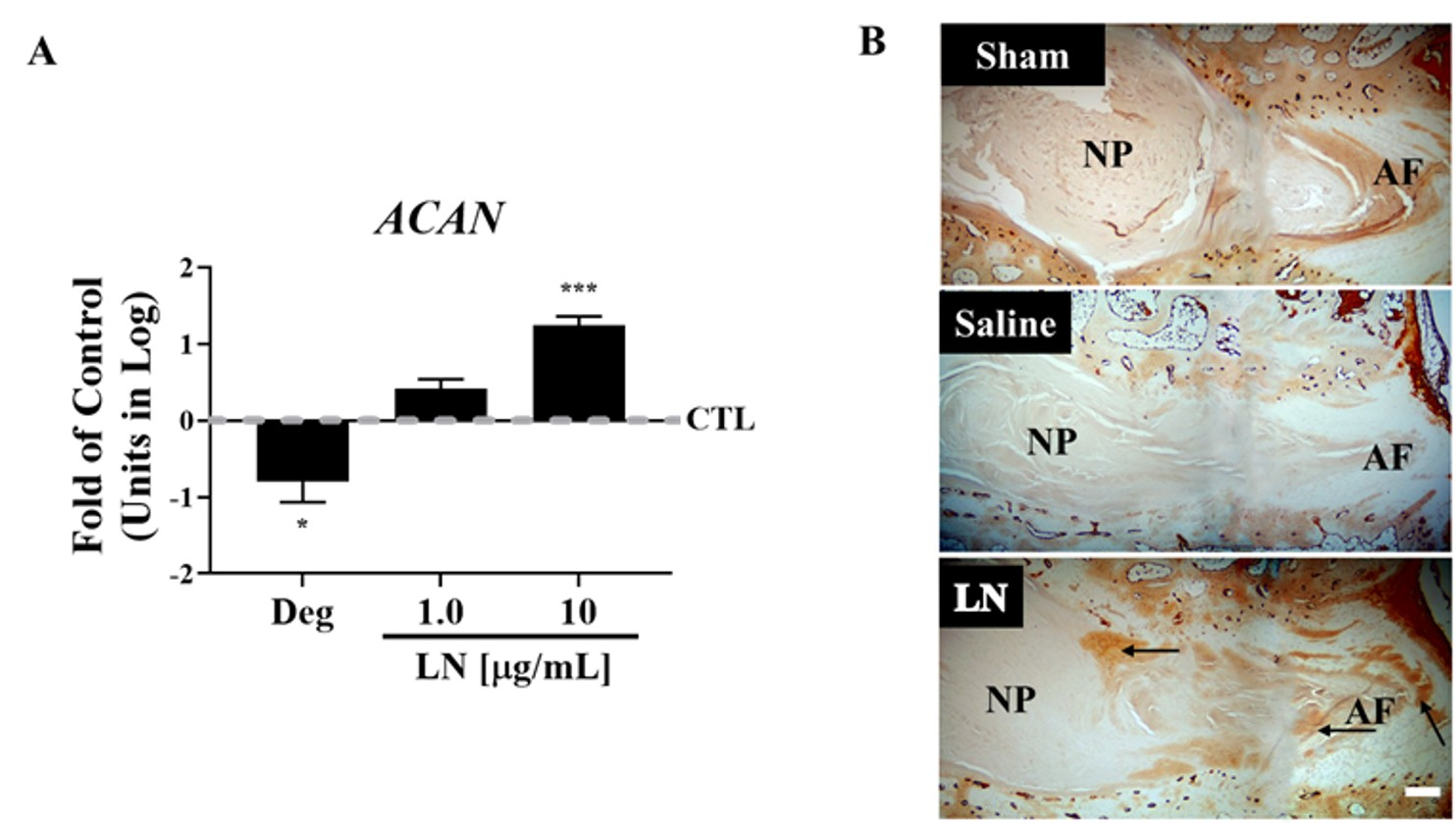

Supplement: Supplementary file 1 [file biomolecules-15-00603-s001.zip › Figure S2.jpg]

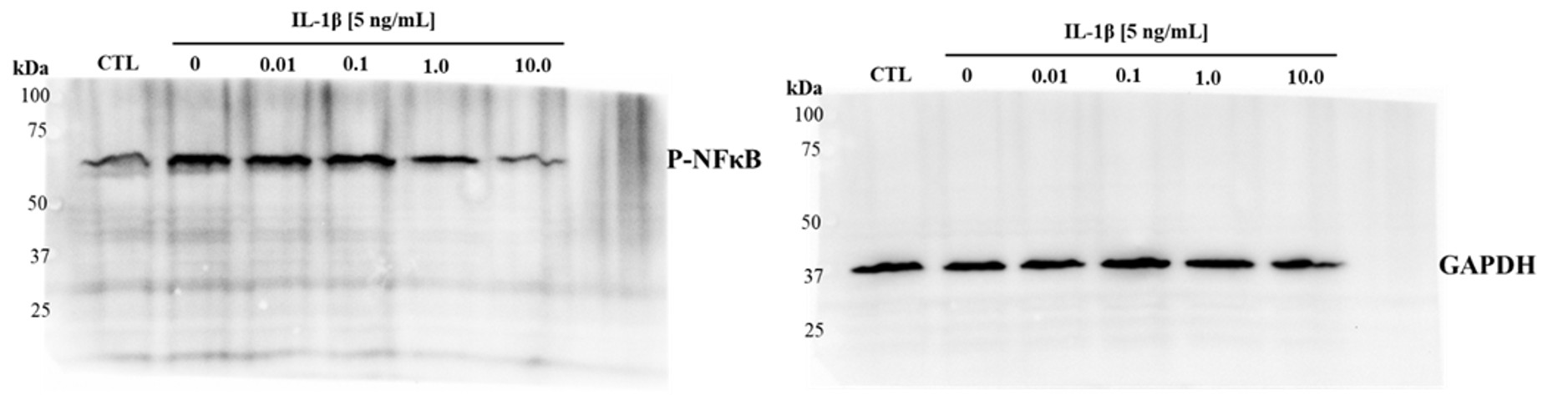

Supplement: Supplementary file 1 [file biomolecules-15-00603-s001.zip › Figure S3.jpg]

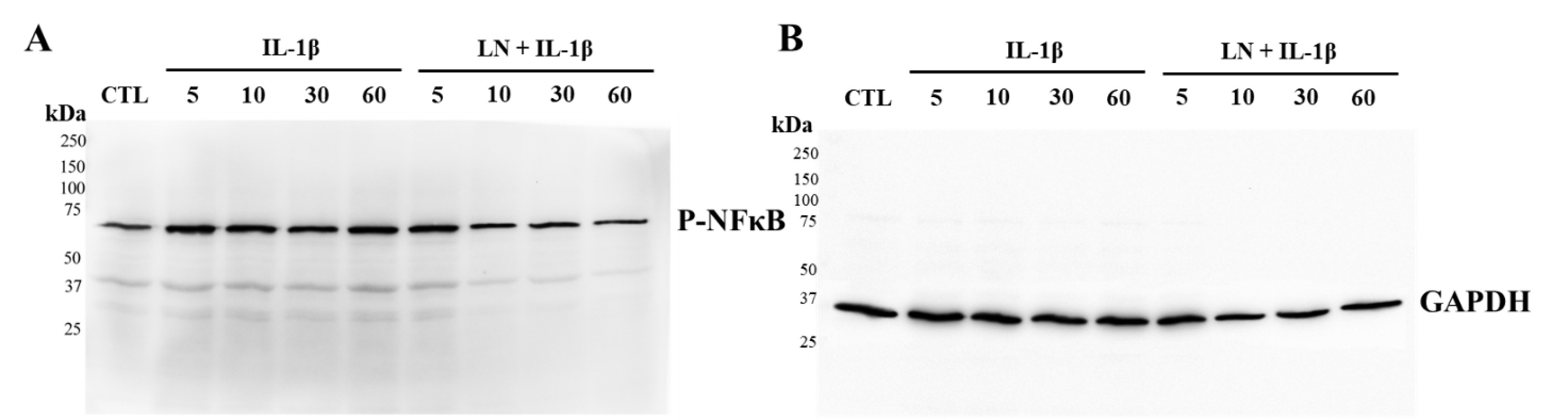

Supplement: Supplementary file 1 [file biomolecules-15-00603-s001.zip › Figure S4.jpg]

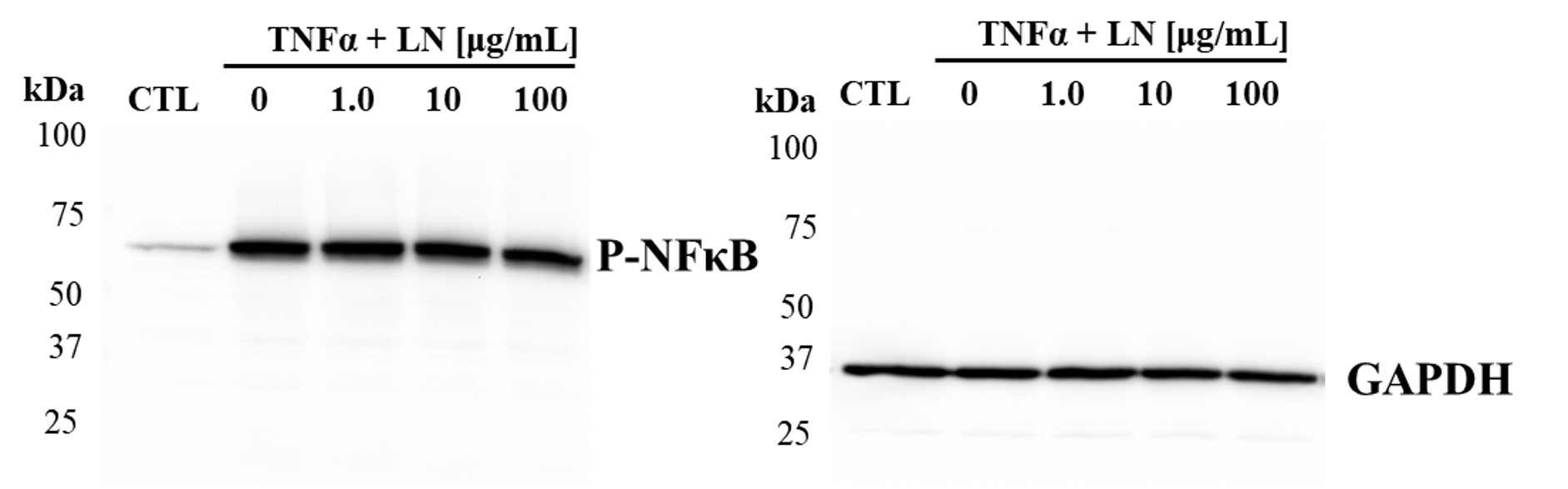

Supplement: Supplementary file 1 [file biomolecules-15-00603-s001.zip › Figure S5.jpg]

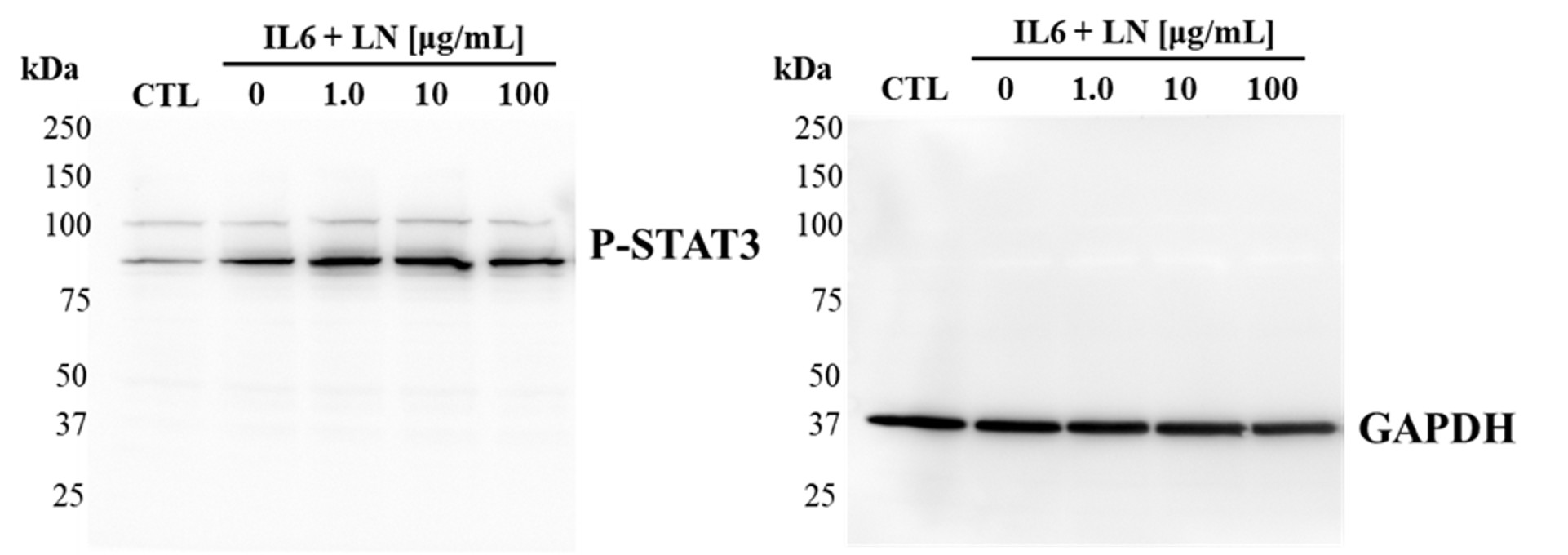

Supplement: Supplementary file 1 [file biomolecules-15-00603-s001.zip › Figure S6.jpg]

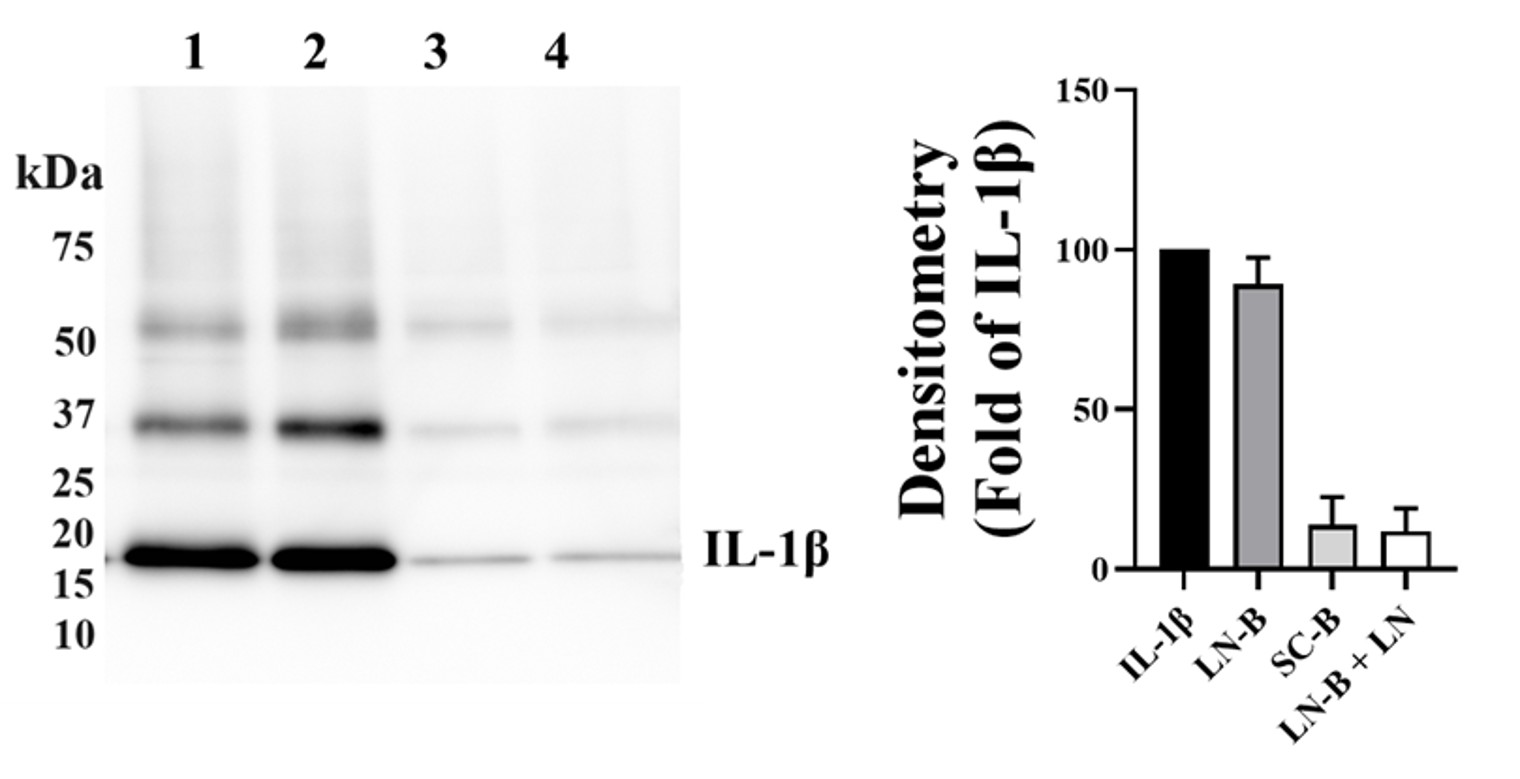

Supplement: Supplementary file 1 [file biomolecules-15-00603-s001.zip › Figure S7.jpg]

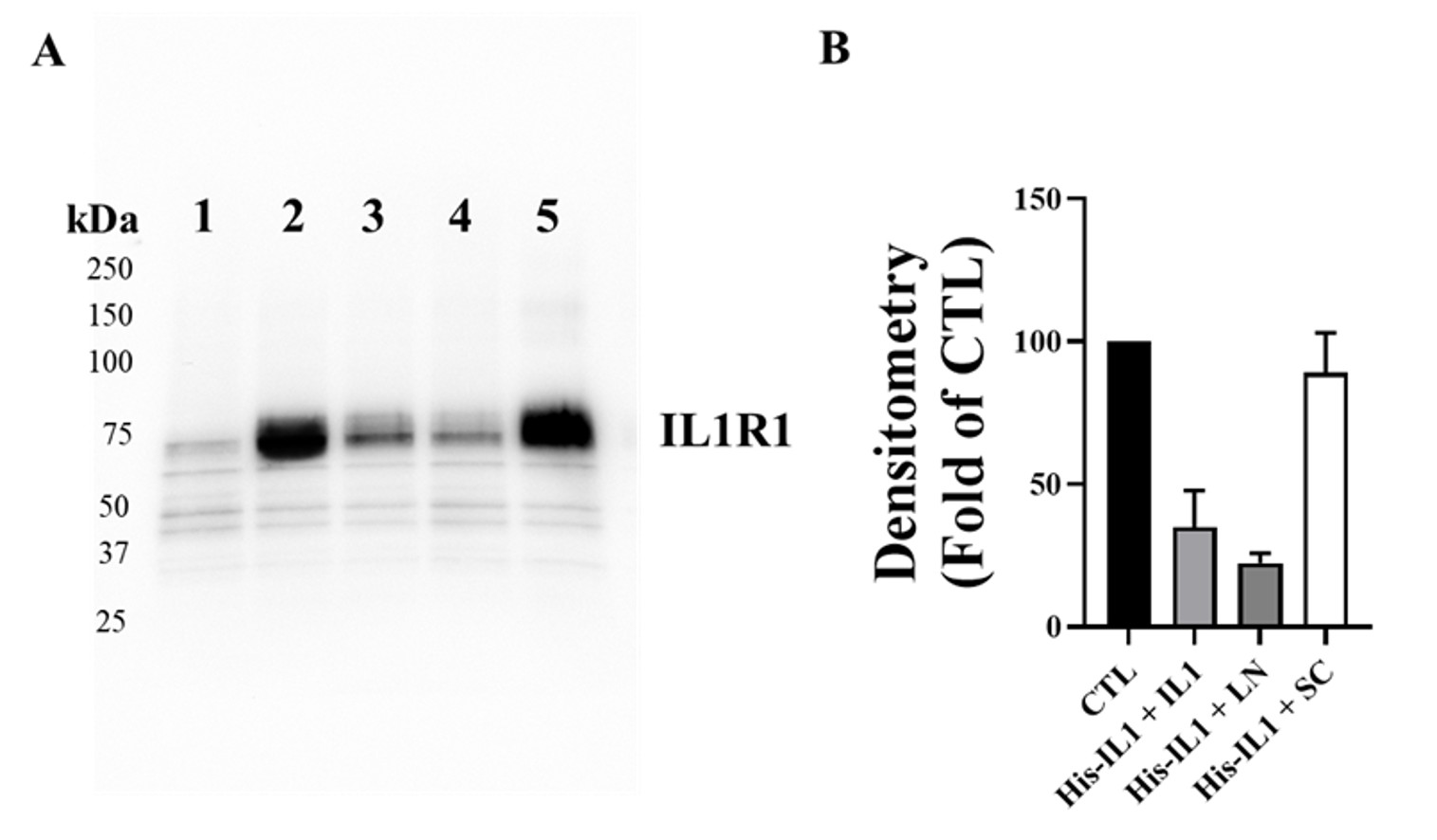

Supplement: Supplementary file 1 [file biomolecules-15-00603-s001.zip › Figure S8.jpg]
